# First Insight into the Seroepidemiology of Hepatitis E Virus (HEV) in Dogs, Cats, Horses, Cattle, Sheep, and Goats from Bulgaria

**DOI:** 10.3390/v15071594

**Published:** 2023-07-21

**Authors:** Ilia Tsachev, Krasimira Gospodinova, Roman Pepovich, Katerina Takova, Todor Kundurzhiev, Gergana Zahmanova, Kristin Kaneva, Magdalena Baymakova

**Affiliations:** 1 Department of Microbiology, Infectious and Parasitic Diseases, Faculty of Veterinary Medicine, Trakia University, 6000 Stara Zagora, Bulgaria; 2 Department of Infectious Pathology, Hygiene, Technology and Control of Foods from Animal Origin, Faculty of Veterinary Medicine, University of Forestry, 1797 Sofia, Bulgaria; 3 Department of Plant Physiology and Molecular Biology, University of Plovdiv, 4000 Plovdiv, Bulgaria; 4 Department of Occupational Medicine, Faculty of Public Health, Medical University, 1527 Sofia, Bulgaria; 5 Department of Technology Transfer and IP Management, Center of Plant Systems Biology and Biotechnology, 4000 Plovdiv, Bulgaria; 6 Department of Infectious Diseases, Military Medical Academy, 1606 Sofia, Bulgaria

**Keywords:** Bulgaria, hepatitis E virus, one health, seroprevalence, zoonoses

## Abstract

In recent years, hepatitis E virus (HEV) infection has been found to be widespread among different animal species worldwide. In Bulgaria, high HEV seropositivity was found among pigs (60.3%), wild boars (40.8%), and East Balkan swine (82.5%). The aim of the present study was to establish the seroprevalence of HEV among dogs, cats, horses, cattle, sheep, and goats in Bulgaria. In total, 720 serum samples from six animal species were randomly collected: dogs—90 samples; cats—90; horses—180; cattle—180; sheep—90; and goats—90. The serum samples were collected from seven districts of the country: Burgas, Kardzhali, Pazardzhik, Plovdiv, Sliven, Smolyan, and Stara Zagora. The animal serum samples were tested for HEV antibodies using the commercial Wantai HEV-Ab ELISA kit (Beijing, China). The overall HEV seroprevalence among different animal species from Bulgaria was as follows: dogs—21.1%; cats—17.7%; horses—8.3%; cattle—7.7%; sheep—32.2%; and goats—24.4%. We found the lowest overall HEV seropositivity in Plovdiv district (6.2%; 4/64; *p* = 0.203) and Smolyan district (8.8%; 4/45; *p* = 0.129), vs. the highest in Pazardzhik district (21.6%; 29/134; *p* = 0.024) and Burgas district (28.8%; 26/90; *p* = 0.062). To the best of our knowledge, this is the first serological evidence of HEV infection in dogs, cats, horses, cattle, sheep, and goats from Bulgaria. We found high HEV seropositivity in small ruminants (sheep and goats), moderate seropositivity in pets (dogs and cats), and a low level of seropositivity in large animals (horses and cattle). Previous Bulgarian studies and the results of this research show that HEV infection is widespread among animals in our country. In this regard, the Bulgarian health authorities must carry out increased surveillance and control of HEV infection among animals in Bulgaria.

## 1. Introduction

Worldwide, the hepatitis E virus (HEV) is one of the leading causes of acute viral hepatitis, transmitted mainly via the fecal-oral route or by contact with infected animals and their products. In recent decades, the understanding of the HEV infection has changed considerably. HEV was first detected in June 1983 by a scientific team led by Mikhail S. Balayan [1]. According to the latest revision of the International Committee on Taxonomy of Viruses (ICTV), HEV belongs to the *Hepeviridae* family, which is divided into two subfamilies: *Orthohepevirinae* and *Parahepevirinae* [2,3]. *Orthohepevirinae* has four genera: *Avihepevirus*, *Chirohepevirus*, *Paslahepevirus,* and *Rocahepevirus* [2]. The hepeviruses from *Paslahepevirus balayani* and *Rocahepevirus ratti* include strains that cause acute hepatitis in humans of zoonotic origin [2,4,5,6]. The *Paslahepevirus balayani* species have been assigned to eight genotypes (gt): HEV gt 1 (Southern Asia; humans), HEV gt 2 (Africa and Mexico; humans), HEV gt 3 and HEV gt 4 (America, Asia, and Europe; humans, pigs, cattle, deer, goats, rabbits, rats, sheep, *Tursiops truncatus*, etc.), HEV gt 5 and HEV gt 6 (Japan; wild boars), HEV gt 7 (United Arab Emirates; *Camelus dromedarius*, humans), and HEV gt 8 (China; *Camelus bactrianus*) [7,8,9,10,11,12,13,14,15,16,17,18]. HEV gt 1 and HEV gt 2 cause hepatitis disease in humans and are transmitted by the fecal-oral route, mainly due to contaminated water and lower sanitary and hygienic standards. Worldwide, HEV gt 3 and HEV gt 4 are found primarily in domestic pigs and wild boar. They can be transferred to humans, usually through the consumption of raw or undercooked infected meat [19]. In addition, transmission to humans, specifically hunters, veterinarians, and forest workers, can also occur as a result of occupational exposure to the virus [20,21]. A patient from the United Arab Emirates was infected with HEV gt 7 as a result of regular consumption of camel milk [14]. Infection with rat HEV (*Rocahepevirus ratti*) was also observed in humans, and evidence of rat HEV was found in animals other than rodents [22,23].

The role and impact of other host species (wild and domestic) in HEV transmission are still under investigation, but a growing number of studies have shown that HEV infections of animals with close contact with humans, including dogs, cats, cattle, horses, goats, and sheep, are also possible [24,25,26,27]. In 2001, Arankalle et al. found IgG anti-HEV antibodies in dogs from Pune, India (22.7%) and cattle from Surat, India (4.4%) and Pune, India (6.9%) [28]. Okamoto et al. detected antibodies to HEV in 135 sera of Japanese pet cats (the A_450_ values of anti-HEV antibodies ranged from 0.026 to 2.917, and 33% of the samples had an A_450_ value of ≥0.600) [29]. The first evidence of HEV in horses was found in Egypt (13%, or 26/200 equine serum samples, were positive by HEV IgG serological assay) [30]. In 1994, Usmanov et al. conducted experimental inoculation of lambs with HEV (a pool of 10% HEV patient fecal suspension containing HEV isolates Osh-225 and Osh-228) and indicated the susceptibility of lambs to this virus [31]. HEV seropositivity in goats was first reported by Zhang et al. [32]. They found that 24.0% of the tested goats from Eastern China were positive for anti-HEV IgG antibodies [32].

Recent studies have shown the spread of HEV and the prevalent genotypes among humans and pigs in Bulgaria. HEV-specific antibodies were found in pigs from farrow-to-finish farms (36.0% and 60.3%) [33,34], wild boars (12.5% and 40.8%) [35,36] and East Balkan swine—the only aboriginal pig breed in Bulgaria (82.5%) [37]. The HEV seroprevalence among pigs and wild boars in Bulgaria is similar to the reported seroprevalence rate in European countries, which ranges between 30 and 100% [38,39,40,41,42,43]. Krumova-Valcheva et al. reported overall 10.8% HEV RNA-positive fecal samples among swine from Bulgarian farrow-to-finish pig farms [44]. HEV RNA detection studies in pigs, carried out in many other European countries, have shown similar results: Slovakia—13.7% [45]; Italy—42.2% [46]; the Netherlands—15.0% [47]; Belgium—7.0% [47]; Spain—18.8% [48]; and Hungary—21.0% [49]. Palombieri et al. reported that HEV gt 3 (subtype C) probably dominated among Bulgarian pigs [50]. Bruni et al. show that the acute hepatitis E human cases in Bulgaria are caused by HEV gt 3 (subtypes 3e, 3f, and 3c), which are usually found in pigs and wild boars, revealing the possible zoonotic transmission of the HEV infection to humans [51]. Until now, HEV has only been detected in pigs in Bulgaria, i.e., there are no data on the spread of the virus among other animal reservoirs in our country. The aim of the present study was to establish the seroprevalence of HEV among dogs, cats, horses, cattle, sheep, and goats in Bulgaria. To the best of our knowledge, this is the first seroepidemiological HEV survey on these animal reservoirs in Bulgaria. In addition, this is the first serological evidence of HEV infection in dogs, horses, cattle, sheep, and goats in Southeastern Europe (Balkan Peninsula).

## 2. Materials and Methods

### 2.1. Study Design and Data Collection

The current research was conducted between 1 June 2022 and 30 December 2022. In total, 720 serum samples from six animal species were randomly collected from randomly chosen farms and animals: dog (*Canis lupus familiaris*)—90 samples; cat (*Felis silvestris catus*)—90; horse (*Equus ferus caballus*)—180; cattle (*Bos primigenius taurus*)—180; sheep (*Ovis aries*)—90; and goat (*Capra hircus*)—90. The animals included in the present study were domestically bred. The samples from the dogs and cats were obtained at the University Veterinary Hospital on different occasions. These animals were pets and were most often kept in urban households. Horses were used as working animals and were bred by minority ethnic communities (with low education) in rural and suburban areas. Equine samples were collected by mobile veterinary teams that visited the minority ethnic communities in their place of residence. Cattle were raised on farms (100–200 animals in a herd). During the routine veterinary examination, the veterinarian took a sample from the cattle. Sheep and goats were kept in herds of 100–200 animals in the rural areas of the administrative districts. The sheep and goat samples were taken during the routine veterinary examination of these animals. For all animals, we received data on which administrative district they live in. Additionally, we collected sex and age information for dogs, cats, and horses.

**Figure 1:**
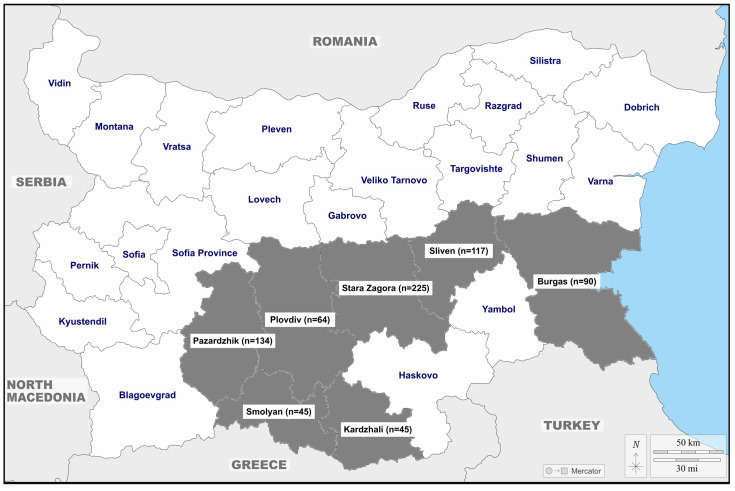
The number of all animal species studied by districts (in gray).

The serum samples were collected from different administrative areas of the country: Burgas district (approximately 27°46′ E Longitude; 42°50′ N Latitude), Kardzhali district (approx. 25°37′ E Long; 41°63′ N Lat), Pazardzhik district (approx. 24°33′ E Long; 42°19′ N Lat), Plovdiv district (approx. 24°74′ E Long; 42°13′ N Lat), Sliven district (approx. 26°32′ E Long; 42°68′ N Lat), Smolyan district (approx. 24°70′ E Long; 41°57′ N Lat), and Stara Zagora district (approx. 25°63′ E Long; 42°42′ N Lat). The number of animals studied by districts is presented in Figure 1.

A blood sample of up to 5 mL was obtained from each animal species. The blood samples were kept in plain vacutainers (without anticoagulant reagent) at room temperature (20.0 °C) until visible clot retraction. After being centrifuged at 1500× *g* for 10 min., the sera were separated and stored at −20.0 °C until analysis. Sample testing and the analysis of serological results were performed at the Laboratory of Animal Infectious Diseases, Faculty of Veterinary Medicine, Trakia University, 6000 Stara Zagora, Bulgaria.

### 2.2. HEV Antibody Detection

The animal serum samples were tested for HEV antibodies using the commercial Wantai HEV-Ab ELISA kit (Beijing Wantai Biological Pharmacy Enterprise Co., Ltd., Beijing, China; Catalog number: WE-7396). The applied serological test was for the qualitative detection of total HEV antibodies in animal serum. It was intended for the diagnosis of HEV infection and seroprevalence studies among different animal species. This commercial ELISA test has been shown to be superior to other ELISA tests for detecting HEV antibodies in previous research (both the Wantai test and DiaPro, Milan, Italy, have shown the highest sensitivity) [52].

### 2.3. Ethical Considerations

Animals were treated humanely according to Bulgarian national legislation. The owners of the animals participating in this study provided “Five freedoms of animal welfare”: freedom from hunger and thirst; freedom from discomfort; freedom from pain, injury, and disease; freedom to express normal behavior; and freedom from fear and distress. Immobilization procedures during the blood collection rule out pain, fear, or agitation in the animals. Sampling was carried out by qualified personnel (veterinarians) who used protective equipment (masks, gloves, glasses, boots, etc.). Written informed consent for participation in the present study was obtained from all animal owners. The current study was permitted and approved by the Local Ethics Committee at Trakia University, Stara Zagora, Bulgaria (FVM-05/17 May 2022).

### 2.4. Statistical Analysis

Data analysis was performed with the help of SPSS Statistics 20.0 (IBM Corp., Armonk, NY, USA) and Excel 2007 (Microsoft, Redmond, WA, USA). The data were entered and arranged in MS Excel. HEV prevalence was estimated from the ratio of positive samples to the total number of samples analyzed, with confidence intervals of 95%. For all characteristics, the Z-test was used to test the hypothesis that the observed proportion is equal to a predetermined proportion. The samples (dogs, cats, and horses) were divided into three age groups and categorized according to sex. Univariate logistic regression was used to assess the risk regarding sex, age, animal species, HEV-positive result, and districts. Multivariate logistic regression was applied to assess the independent influence of animal species on sex and age. A *p*-value < 0.05 was considered statistically significant.

## 3. Results

In total, 15.9% (115/720) anti-HEV-positive samples were detected among all animals (Table 1). The overall HEV seroprevalence among different animal species from Bulgaria was as follows: dogs—21.1% (19/90); cats—17.7% (16/90); horses—8.3% (15/180); cattle—7.7% (14/180); sheep—32.2% (29/90); and goats—24.4% (22/90).

The samples from dogs, cats, and horses were categorized according to sex. Regarding sex, a higher HEV seropositivity was found in the female dogs (26.0%), followed by the male cats (20.4%), male dogs (15.9%), and female cats (15.2%), but the observed difference is not statistically significant (Table 2). In horses, we observed a statistically significantly lower rate of HEV antibodies in females (4.1%) compared to males (13.4%) (odds ratio, OR = 0.243; *p* = 0.029). Data on HEV seroprevalence were collected according to age groups that were each divided into three different age groups (animals < 2 years old; 2–6 years old; >6 years old). The research shows that the anti-HEV antibody prevalence percentage in age group <2 years old animal species was 14.5% (7/48); age group 2–6 years old—13.6% (19/139); and age group > 6 years old—13.8% (24/173) (*p* = 0.988) (Table 2). There was no significant difference among the age groups. 

HEV seroprevalence distribution varies by region (Table 3 and Table 4). We detected the highest HEV-positive results for horses in the Sliven district (9.4%); cattle in the Stara Zagora district (13.3%); sheep in the Pazardzhik district (33.3%); and goats in the Burgas district (26.6%) (Table 3). In addition, we found that the districts with the highest HEV seropositivity were Burgas (28.8%) and Pazardzhik (21.6%); vice versa, the districts with the lowest HEV seropositivity were Kardzhali (0.0%) and Plovdiv (6.2%) (Table 4). The binary logistic regression showed that the risk of a HEV-positive result was 20.469 times higher for Stara Zagora district (*p* = 0.035), 25.446 times higher for Pazardzhik district (*p* = 0.024), and 14.660 times higher for Burgas district (*p* = 0.062) compared to the reference district of Kardzhali.

## 4. Discussion

Numerous studies have shown animal transmission of HEV disease to humans and have identified a wide variety of animal species that can act as HEV hosts [7,8,10,19]. Many of these studies are focused on HEV in pigs and wild boars [11,33,34,35,36,37,38,39,40]. However, it appears that other animal species, which are in much closer contact with humans, also have high HEV seropositive rate. In this research, we report HEV seropositivity among a wide group of animals (dogs, cats, horses, cattle, sheep, and goats), and it is the first seroepidemiological HEV survey of this kind in Bulgaria. The overall HEV seroprevalence was 21.1% in dogs, 17.7% in cats, 8.3% in horses, 7.7% in cattle, 32.2% in sheep, and 24.4% in goats.

The reported HEV seroprevalence in dogs in European countries varies widely. What we have observed fits somewhat in the middle. The HEV seropositivity in dogs in Bulgaria was slightly higher than that reported in the Netherlands (18.5%) [53] and significantly higher than those those reported in the United Kingdom (0.8%) [54], Italy (5.0%) [55], and Spain (9.9%) [24]. The HEV seroprevalence observed in Switzerland (38.0%) [56] and Germany (56.5%) [57], however, was significantly higher than what we observed. The seroprevalence found in our cats was 17.7%, which is approximately at a mean level compared to the results of other European countries: Spain (2.8%) [24], and (37.0%) [58]; Italy (3.1%) [59]; Turkey (5.4%) [60]; Netherlands (14.8%) [53]; and Germany (32.3%) [57]. Although it is difficult to make a comparison between different studies due to the use of different serological tests and a difference in the number of animals studied, we can state that the seroprevalence of HEV in cats and dogs in Bulgaria should be considered moderate.

Several studies have proposed HEV serological spread in animals according to sex and age. Caballero-Gomez et al. reported 12.4% HEV seropositivity in male urban dogs compared to 6.4% in females [24]. German authors showed 47.2% HEV-positive results in male cats from Brandenburg and 27.6% in female cats [57]. Capozza et al. reported 3.9% HEV seropositivity in male household cats compared to 2.0% in females [59]. Some surveys established an equable distribution of HEV seroprevalence among different age groups. Spanish authors found 11.1% and 0.0% HEV seropositivity in yearling dogs and cats, respectively. HEV seropositivity of 7.9% and 0.0% in sub-adult dogs and cats, respectively. Finally, in adult dogs and cats, the HEV seropositivity was 10.8% and 3.9%, respectively [24]. Bernardini et al. reported 0.0% HEV-positive results in dogs under 2 years old, 6.9% in dogs between 2 and 5 years old, and 3.7% in dogs over 5 years of age [55]. Italian researchers found 0.0% (<2 years old) HEV seropositivity in household cats; 4.4% in cats aged 2–8 years, 4.6% in cats aged 9–14 years, 0.0% in cats >14 years or of an undetermined age [59]. Cagirgan et al. reported 3.2% HEV positivity in domestic cats (0–2 age group); 1.09% in cats of the 2–8 age group, and 1.09% in those older than 8 years [60]. Our overall HEV seropositivity in dogs (21.1%) and cats (17.7%) is similar to that of Li et al. from the Netherlands, who reported 18.5% among dogs and 14.8% in cats [53].

There are various potential reasons for the moderate HEV seroprevalence among pets (dogs and cats) presented by our study. For example, the animals’ behavior could influence the seropositivity. During the walk, dogs like to sniff and dig in the bushes, grass, and garbage. So, this instinctual behavior could increase the risk of HEV contamination. In addition, cats love to hunt small mammals such as rats and mice, which could be a serious prerequisite for HEV transmission. In this regard, Caballero-Gomez et al. suggested that both HEV gt 3 (*Paslahepevirus balayani*) and rat HEV-C1 (*Rocahepevirus ratti*) genotypes circulate in urban cats and dogs in Spain, which hypothesis supports the above-mentioned ways for infection of pets [24]. Dogs and cats are carnivores and it is possible that they can become infected via the food chain by consuming raw meat contaminated with swine HEV gt 3 or rat HEV-C1. Furthermore, the moderate HEV seropositivity in our dogs (21.1%) and cats (17.7%) is similar to that found among Bulgarian blood donors (25.9%) [20] and individuals with Guillain-Barre syndrome (24.5%) [61].

Most equine HEV studies show low seropositivity. Saad et al. presented 13% HEV IgG ELISA positive results among privately owned work horses from suburban areas of Cairo (Egypt), particularly “Old Cairo” [30]. Low HEV seropositivity among horses was also found in other countries: China (11.0%) [62] and (16.3%) [32]; South Korea (12.4%) [63]; and the Netherlands (18.18%) [53]. Yoon et al. reported a higher HEV seroprevalence in male horses (16.7%) compared to females (11.7%) [63]. The same authors reported different results for different age groups: 1–2 years (20.0%); 3–10 years (9.9%); >10 years (13.1%) [63]. Our results in horses (8.3%) are similar to those of Fu et al. from Xinjiang, China, who reported 11.0% HEV-positive results in domestic horses [62].

The prevalence of HEV in cattle varies widely in different countries around the world. In Croatia, 0.0% HEV seropositivity was found among cattle (*Bos taurus*) [64]; Spain (0.0%) [58]; South Korea (0.0%) [65]; Brazil (1.42%) [66]; India (4.4%) in Surat and (6.9%) in Pune [28]; China (6.0%) [32], (6.3%) [67], (6.5%) [62], (18.7%) [68], (28.2%) [69], (29.35%) [70]; Jordan (14.5%) [71]; USA (20.4%) [72]; and Egypt (21.6%) [73]. Our overall HEV seropositivity in cattle (7.7%) is similar to that of Arankalle et al. from Pune, Western India, who reported (6.9%) in cattle [28].

We found low levels of HEV antibodies in large animals (horses (8.3%); cattle (7.7%)). The most likely reason could be the horse’s feeding by minority ethnic communities. Usually, these population groups have low education and low social status. They keep the horses alone (one or two horses per household). In addition, these horses are bred in rural and suburban areas. Their habitats do not interact with the habitats of the main HEV reservoirs, such as those of wild boar or deer. A potential reason for the low HEV seropositivity in cattle could be the controlled, balanced diet and the lack of contact with free pastures and meadows. These factors reduce the risk of HEV exposure and, consequently, high HEV seroprevalence.

The prevalence of HEV infection in sheep and goats varies widely between studies. Low seropositivity was found in sheep in Spain (2.1%) [74] and (2.6%) [58]—compared to high seropositivity reported in Portugal (16.6%) [75]; and Italy (21.3%) [76] and (21.6%) [77]. HEV antibodies were detected in goat sera from Spain (1.6%) [58] and (13.8%) [74]; Italy (11.4%) [77]; and the USA (16%) [78]. Our results for sheep (32.2%) and goats (24.4%) are similar to those of Palombieri et al. from Italy, who reported (21.6%) HEV-positive results in sheep serum samples [77], and Sanford et al. from the USA, who presented 16% HEV-positive results in serum samples of mature goats [78].

We found high HEV seropositivity among small ruminants (sheep, 32.2%; goats, 24.4%). These animals are bred in rural areas of our country. In most cases, these sheep and goats graze on free pastures and meadows in areas outside the settlements. Frequently, these animals drink water from natural springs and untested sources. These factors lead to an increased risk of HEV infection. On the other hand, the high HEV seropositivity among our small ruminants is similar to that found in Bulgarian wild boars (40.8%) [36] and East Balkan swine—the only aboriginal pig breed in Bulgaria (82.5%) [37].

The serological test used could be a potential reason for different HEV-positive results between different seroepidemiological surveys. We used the commercial Wantai HEV-Ab ELISA kit (Beijing Wantai Biological Pharmacy Enterprise Co., Ltd., Beijing, China). In fact, it is the most widely used serological test for HEV infection in animals [32,53,59,62,63,69,70,72,75,76,77]. Additionally, Norder et al. reported that this commercial ELISA test (produced by Wantai, Beijing, China) has been shown to be superior to other ELISA tests for detecting HEV antibodies [52]. In this regard, we could assume that our results are adequate to reality and in unison with our results from other animal and human studies.

It is well known from the scientific literature that male animals are more likely to be affected by HEV [24,57,59,63,73,79,80], and the incidence of HEV infection increased with age [13,24,79,81]. Unfortunately, in some studies, this information was missing. In this regard, in part of the studies on the prevalence of HEV infection in animal species, it is not possible to analyze the influence of sex and age. Furthermore, it is well known that there is an age-dependent trend for domestic pigs and wild boars—with increasing age = increasing HEV-positive results [33,34,37]. Therefore, it is good to have data on these two important indicators (sex and age).

The present study has some limitations that need to be addressed. First, we did not perform molecular analysis of HEV (HEV RNA testing) due to technical and financial reasons. Second, it was not a nationwide survey, i.e., the present research showed data on several districts in our country. Third, we had sex and age information only for some animals (dogs, cats, and horses). Fourth, information on herds (for cattle, sheep, and goats) was not collected. Fifth, the comparison of HEV seropositivity by administrative districts in our survey should be interpreted with care and kept in mind that individual districts include different animal species, i.e., comparison of different animal populations in different districts. Sixth, in the absence of a demonstration of HEV RNA, the epidemiological role of all investigated animal species in relation to the risk of transmission to humans remains to be clarified. In this regard, the results and conclusions should be interpreted with caution. Despite these limitations, this research has its merits. To the best of our knowledge, this is the first seroepidemiological HEV survey among dogs, cats, horses, cattle, sheep, and goats in Bulgaria. Furthermore, this is the first serological evidence of HEV infection in dogs, horses, cattle, sheep, and goats from Southeastern Europe (the Balkan Peninsula).

## 5. Conclusions

We conducted a seroepidemiological study on HEV infection in animal species from Bulgaria. We found high HEV seropositivity in small ruminants (sheep—32.2%; goats—24.4%); moderate in pets (dogs—21.1%; cats—17.7%); and lower in large animals (horses—8.3%; cattle—7.7%). These results enhance our knowledge about the prevalence of HEV infection in animals from Bulgaria. Both previous Bulgarian studies [33,34,35,36,37,44,50] and the current results of this research show that HEV infection is widespread among animals in our country. Furthermore, HEV seropositivity is widespread among some groups of the Bulgarian population: blood donors (25.9%) [20], general hunters (48.7%) [20], hunters of wild boars (51.6%) [20], and individuals with Guillain-Barre syndrome (24.5%) [61]. These facts are a serious reason for increasing surveillance and control of HEV infection among animals and humans in Bulgaria. In this regard, all Bulgarian health authorities and scientific organizations are recommended to improve their policies and programs for the prevention and control of HEV infection in our country.

## Figures and Tables

**Table 1:** Seroprevalence of hepatitis E virus (HEV) infection in different species from Bulgaria.

Species	Investigated Animals, n	HEV-Positive		95% CI	
		Number	Percent	Lower	Upper
Dog	90	19	21.1	13.2	30.9
Cat	90	16	17.7	10.5	27.2
Horse	180	15	8.3	4.7	13.3
Cattle	180	14	7.7	4.3	12.6
Sheep	90	29	32.2	22.7	42.9
Goat	90	22	24.4	15.9	34.6
Total	720	115	15.9	13.3	18.8

Note: CI—confidence interval.

**Table 2:** Univariate and multivariable analysis showing the association between HEV seropositivity and different variables (sex and age).

Animal Species		Variable	Total	HEV-Positive, n (%)	HEV-Negative, n (%)	Univariate Logistic Regression			Multivariable Logistic Regression		
						OR	95% CI	*p*-Value	OR	95% CI	*p*-Value
Dog	Sex	Male	44	7 (15.9)	37 (84.1)	1.00			1.00		
		Female	46	12 (26.0)	34 (74.0)	1.86	0.65–5.28	0.241	2.09	0.71–6.14	0.177
	Age	<2 years old	20	3 (15.0)	17 (85.0)	1.00			1.00		
		2–6 years old	30	7 (23.3)	23 (76.7)	1.72	0.38–7.65	0.474	1.95	0.43–8.87	0.385
		>6 years old	40	9 (22.5)	31 (77.5)	1.64	0.39–6.90	0.496	2.05	0.46–9.06	0.339
Cat	Sex	Male	44	9 (20.4)	35 (79.6)	1.00			1.00		
		Female	46	7 (15.2)	39 (84.8)	0.69	0.23–2.07	0.517	0.62	0.19–1.99	0.424
	Age	<2 years old	14	3 (21.4)	11 (78.6)	1.00			1.00		
		2–6 years old	37	6 (16.2)	31 (83.8)	0.71	0.15–3.33	0.664	0.58	0.11–2.98	0.515
		>6 years old	39	7 (17.9)	32 (82.1)	0.80	0.17–3.65	0.776	0.65	0.12–3.26	0.601
Horse	Sex	Male	82	11 (13.4)	71 (86.6)	1.00			1.00		
		Female	98	4 (4.1)	94 (95.9)	0.27	0.08–0.89	0.033	0.24	0.06–0.86	0.029
	Age	<2 years old	14	1 (7.1)	13 (92.9)	1.00			1.00		
		2–6 years old	72	6 (8.3)	66 (91.7)	1.18	0.13–10.65	0.882	0.67	0.06–6.78	0.739
		>6 years old	94	8 (8.5)	86 (91.5)	1.20	0.14–10.47	0.863	0.53	0.05–5.44	0.593

Note: OR—odds ratio.

**Table 3:** Serological evidence of HEV infection in different districts of Bulgaria.

Species by District	Investigated Animals, n	HEV-Positive		95% CI	
		Number	Percent	Lower	Upper
Stara Zagora district					
Dog	90	19	21.1	13.2	30.9
Cat	90	16	17.7	10.5	27.2
Cattle	45	6	13.3	5.0	26.8
Pazardzhik district					
Horse	44	4	9.0	2.9	21.6
Sheep	45	15	33.3	19.9	48.9
Goat	45	10	22.2	11.2	37.1
Burgas district					
Sheep	45	14	31.1	18.2	46.6
Goat	45	12	26.6	14.6	41.9
Sliven district					
Horse	117	11	9.4	4.8	16.2
Plovdiv district					
Horse	19	0	0.0	NA	NA
Cattle	45	4	8.8	2.4	21.1
Smolyan district					
Cattle	45	4	8.8	2.4	21.1
Kardzhali district					
Cattle	45	0	0.0	NA	NA

Note: NA—not applicable.

**Table 4:** Binary logistic regression showing the association between HEV seropositivity and different districts in the country.

District	Investigated Animals, n	HEV-Positive, n (%)	HEV-Negative, n (%)	Binary Logistic Regression		
				OR	95% CI	*p*-Value
Kardzhali	45	0 (0.0)	45 (100.0)	1.00		
Plovdiv	64	4 (6.2)	60 (93.8)	6.76	0.35–128.93	0.203
Smolyan	45	4 (8.8)	41 (91.2)	9.85	0.51–188.89	0.129
Sliven	117	11 (9.4)	106 (90.6)	9.82	0.56–170.33	0.116
Stara Zagora	225	41 (18.2)	184 (81.8)	20.46	1.23–339.07	0.035
Pazardzhik	134	29 (21.6)	105 (78.4)	25.44	1.52–425.52	0.024
Burgas	90	26 (28.8)	64 (71.2)	14.66	0.87–245.22	0.062

## Data Availability

Not applicable.
